# Advances in Neonatal Resuscitation and Intensive Care–Contribution of the Special Issue in Neonatal Care

**DOI:** 10.3390/children13101388

**Published:** 2026-10-13

**Authors:** Dharmesh Shah, Rajesh Maheshwari, Pranav Jani

**Affiliations:** 1 Neonatal Intensive Care Unit, Westmead Hospital, Sydney, NSW 2145, Australia; rajesh.maheshwari@health.nsw.gov.au (R.M.); pranav.jani@health.nsw.gov.au (P.J.); 2 Faculty of Medicine and Health, Sydney Medical School, The University of Sydney, Sydney, NSW 2006, Australia

Neonatal intensive care is a recent specialty, with the first neonatal intensive care unit starting in 1960 [1]. The field has advanced remarkably rapidly since then, with significant amounts of research contributing to the practice of evidence-based medicine. This has led to significantly improved survival rates for extremely preterm infants with the lower limits of gestation at which resuscitation is being attempted moving downwards [2]. In addition, there have been remarkable advancements in neonatal resuscitation in the last two decades, and guidelines on neonatal resuscitation keep evolving with this evidence [3].

In this issue of *Children*, the authors have contributed to generating new knowledge about neonatal resuscitation and intensive care. The studies are a mixture of animal studies, manikin studies, systematic reviews, human RCTs and other retrospective and prospective studies. We hope this will help in refining future intensive care and lead to more studies answering emerging questions.

Umbilical cord management has been studied extensively in recent years, with good evidence that delayed cord clamping reduces mortality and other adverse outcomes in preterm infants [4]. As the initial trials on delayed cord clamping excluded infants who needed immediate resuscitation, the management of umbilical cord in such infants cannot be extrapolated from earlier studies. Umbilical cord milking was shown to be harmful in extremely preterm infants [5]. Bawa et al. have compared different durations of delay in clamping the cord in an asphyxiated animal model with simultaneous support of the ventilation. The findings suggest that a delay of 5 min with ongoing ventilation may offer advantages in asphyxiated preterm animals. RCTs in human preterm infants will be required to confirm these findings.

Assessment of heart rate during resuscitation and optimal method for providing chest compressions remains an active area of research. Two studies in this issue throw some light on techniques of chest compression which may be beneficial. Large human studies will be needed to confirm these findings. The use of ECG to monitor heart rate during resuscitation was explored in another RCT, but the study had to be terminated prematurely due to resistance to video recording of the resuscitation, reducing the power of the study.

This issue also addresses various aspects of neonatal intensive care, such as use of lung ultrasound to guide surfactant replacement therapy, reducing complications of central venous access (both umbilical venous catheter and peripherally inserted central catheters) and follow-up of acute kidney injury in NICU graduates. We believe lung ultrasound has an important role to play to optimize the use of surfactants in preterm infants. Its role is likely even more important for lower- and middle-income countries due to the cost of surfactant therapy.

Li et al. have published on an exciting protocol for a prospective cohort study to see if there is a causal relationship between gut microbial colonization and neurodevelopment in preterm infants. If a relationship between gut microbial colonization and neurodevelopment is confirmed, the role of antimicrobial stewardship in NICUs will become even more important.

The systematic review by Mattersberger et al. on acid-base status and its relationship with cerebral oxygenation shows no clear correlation. Cerebral oxygenation is a complex measure with multiple influencing factors such as intravascular volume, cardiac output, hemoglobin and peripheral oxygen saturation. This was elegantly represented in their systematic review, with variable findings regarding correlation of acid-base status with cerebral oxygenation.

Finally, it is important to reflect on the fact that we as clinicians look after not only the sick neonate but also the family unit. Neonatal intensive care is a stressful experience for the parents, and neonates born at the edge of viability can stay in the NICU for months. Bertoncelli et al. present the results of their qualitative study of the impact of neonatal intensive care on the parents and suggest useful strategies that health professionals can utilize to reduce parental stress.

We hope the readers enjoy reading the articles presented in this issue and that this then stimulates future studies to improve the care of newborn infants.

## Data Availability

No new data were created or analyzed in this study. Data sharing is not applicable to this article.

## References

[1] Lussky R.C., Cifuentes R.F., Siddappa A.M. (2005). A history of neonatal medicine—Past accomplishments, lessons learned and future challenges. Part 1—The first century. J. Pediatr. Pharmacol. Ther..

[2] Hyland R.M., Mat H.D., Boly T.J., Thomas B.J., Stanford A.H., Harmon H.M., Bermick J.R., Davila R.C., Colaizy T.T., Dagle J.M. (2025). Outcomes of infants born at 21 weeks’ gestational age. JAMA Netw. Open.

[3] Liley H.G., Weiner G.M., Chair V., Wyckoff M.H., Rabi Y., Schmolzer G.M., de Almeida M.F., Costa-Nobre D., Davis P.G., Dawson J.A. (2025). Neonatal life support: 2025 International liaison committee on resuscitation consensus on science with treatment recommendations. Circulation.

[4] Seidler A.L., Aberoumand M., Hunter K.E., Barba A., Libesman S., Williams J.G., Shrestha N., Aagerup J., Sotiropoulos J.X., Montgomery A.A. (2023). Deferred cord clamping, cord milking and immediate cord clamping at preterm birth: A systematic review and individual participant data meta-analysis. Lancet.

[5] Katheria A., Reister F., Essers J., Mendler M., Hummler H., Subramaniam A., Carlo W., Tita A., Truong G., Davis-Nelson S. (2019). Association of umbilical cord milking vs delayed umbilical cord clamping with death or severe intraventricular hemorrhage among preterm infants. JAMA.

